# The Glycosylphosphatidylinositol-Anchored Superoxide Dismutase of *Scedosporium apiospermum* Protects the Conidia from Oxidative Stress

**DOI:** 10.3390/jof7070575

**Published:** 2021-07-19

**Authors:** Cindy Staerck, Hajar Yaakoub, Patrick Vandeputte, Julie Tabiasco, Charlotte Godon, Amandine Gastebois, Sandrine Giraud, Thomas Guillemette, Alphonse Calenda, Yves Delneste, Maxime Fleury, Jean-Philippe Bouchara

**Affiliations:** 1 Université d’Angers, Université de Bretagne Occidentale, CHU Angers, Groupe d’Etude des Interactions Hôte-Pathogène (GEIHP, EA3142), SFR ICAT, F-49000 Angers, France; staerck.cindy@gmail.com (C.S.); hajar-yaakoub@hotmail.com (H.Y.); pvandepu@gmail.com (P.V.); charlotte.godon@univ-angers.fr (C.G.); amandine.gastebois@univ-angers.fr (A.G.); sandrine.giraud@univ-angers.fr (S.G.); alphonse.calenda@univ-angers.fr (A.C.); mjj.fleury@gmail.com (M.F.); 2 Université d’Angers, Université de Nantes, CHU Angers, Inserm, CRCINA, SFR ICAT, F-49000 Angers, France; julie.tabiasco@inserm.fr (J.T.); yves.delneste@univ-angers.fr (Y.D.); 3 Université d’Angers, Institut Agro, INRAE, IRHS, SFR QUASAV, F-49000 Angers, France; thomas.guillemette@univ-angers.fr

**Keywords:** *Scedosporium apiospermum*, oxidative stress, ROS, GPI-anchored superoxide dismutase, intracellular killing

## Abstract

*Scedosporium* species are common fungal pathogens in patients with cystic fibrosis (CF). To colonize the CF lungs, fungi must cope with the host immune response, especially the reactive oxygen species (ROS) released by phagocytic cells. To this aim, pathogens have developed various antioxidant systems, including superoxide dismutases (SODs) which constitute the first-line protection against oxidative stress. Interestingly*,* one of the *S. apiospermum* SOD-encoding genes (*SODD* gene) exhibits a glycosylphosphatidylinositol (GPI) anchor-binding site and encodes a conidial-specific surface SOD. In this study, a *SODDΔ* mutant was engineered from a non-homologous end joining-deficient strain (*KU70Δ*) of *S. apiospermum*. Compared to its parent strain, the double mutant *KU70Δ/SODDΔ* exhibited increased susceptibility to various oxidizing agents and triazole antifungals. In addition, the loss of SodD resulted in an increased intracellular killing of the conidia by M1 macrophages derived from human blood monocytes, suggesting the involvement of this superoxide dismutase in the evasion to the host defenses. Nevertheless, one cannot disregard an indirect role of the enzyme in the synthesis or assembly of the cell wall components since transmission electron microscopic analysis revealed a thickening of the inner cell wall layer of the conidia. Further studies are needed to confirm the role of this enzyme in the pathogenesis of *Scedosporium* infections, including the production of a recombinant protein and study of its protective effect against the infection in a mouse model of scedosporiosis.

## 1. Introduction

*Scedosporium* species (phylum Ascomycota, order Microascales) are environmental molds usually living as saprophytes, mainly in highly polluted soils and contaminated water [1]. Nevertheless, these fungi may also cause a wide variety of infections in humans. Besides localized infections such as subcutaneous mycetomas or bone and joint infections resulting from traumatic inoculation of some soil fungal elements, pulmonary infections are also described, which are thought to be due to the inhalation of some airborne conidia [2]. Especially, these species have been receiving increasing attention during the past two decades because of their worldwide recognition as significant pathogens in patients with cystic fibrosis (CF). Indeed, *Scedosporium* species rank second among the filamentous fungi colonizing the airways of CF patients, after *Aspergillus fumigatus*, with a prevalence rate ranging from 3.1% to 11.9% [3,4,5,6,7,8,9,10]. In this clinical context, these fungi are usually responsible for a chronic colonization of the airways [11,12], which has been demonstrated as a risk factor for a disseminated infection in the case of lung or heart-lung transplantation [13].

To be able to colonize the respiratory tract, fungi need to evade the host immune attack. Macrophages and neutrophils are the central effector cells of the innate immune system since they release various antimicrobial compounds during the inflammatory reaction triggered by the pathogens, including reactive oxygen and nitrogen species (ROS and RNS) (for a review, see [14]). The key role of ROS in the defense against respiratory pathogens can be illustrated by the frequent occurrence of bacterial or fungal respiratory infections in chronic granulomatous disease, which results from mutations in one of the genes encoding proteins of the mitochondrial NADPH oxidase complex, leading to a defect in the production of superoxide anions (O_2_^●−^) by macrophages and neutrophils [15]. The ability of some pathogens to avoid the oxidative and nitrosative-related damage is mainly due to them being equipped with a variety of enzymatic antioxidants, including superoxide dismutases (SODs), which catalyze the conversion of O_2_^●−^ to hydrogen peroxide (H_2_O_2_) and therefore constitutes the first line of protection of the pathogens against the inflammatory reaction [14,16,17]. This is particularly true in the CF lungs, which are characterized by an exacerbated inflammatory reaction, because of the common colonization of the airways by various microorganisms and the repeated pulmonary exacerbations [18].

Nevertheless, despite the growing interest in *Scedosporium* species, little is known about their pathogenic mechanisms. Only four putative virulence factors have been purified and characterized so far, i.e., a secreted serine-protease [19], a cytosolic Cu,Zn-SOD called SodC [20], the monofunctional catalase CatA1 [21], and the extracellular siderophore *N*^α^-methylcoprogen B which is involved in iron uptake [22,23]. Genes encoding the last two proteins were sequenced, and it was demonstrated that *CatA1* gene was highly expressed in response to oxidative stress [24]. With the availability of the whole genome sequence of *Scedosporium apiospermum* [25], one of the major pathogenic *Scedosporium* species in CF [12], a genome-wide analysis recently conducted permitted the identification of 33 genes encoding proteins putatively involved in ROS or RNS detoxification [26]. Some of these genes, particularly two genes encoding thioredoxin reductases and, to a lesser extent, those encoding a peroxiredoxin and one of the fungal catalases, were shown to be overexpressed upon exposure of 24-h-old fungal hyphae to various chemically-induced oxidative stresses or in co-cultures with activated phagocytic cells [27]. However, because of our experimental procedure, the expression of genes encoding conidial-specific proteins could not be investigated. For instance, among the 33 genes identified by genome mining, one encoding a putative Cu,Zn-SOD (*SODD* gene*;* SAPIO_CDS3212) exhibits a signal peptide and a glycosylphosphatidylinositol (GPI) anchor-binding site [26], and a comparative proteomic analysis of conidia and germ tubes focusing on GPI-anchored cell wall proteins revealed the presence of the corresponding protein exclusively at the conidial surface [28]. Although little information is available regarding fungal GPI-anchored cell wall SODs and their role in pathogenesis, *S. apiospermum* SodD could play an important role in CF in the tug-of-war between the host immune defenses and the fungus since it is likely that conidia constitute the infecting form of the fungus and are therefore the first morphological stage in contact with the host immune defenses.

In this paper, we aimed to define the contribution of the unique GPI-anchored SOD of *S. apiospermum* in evasion to oxidative stress by disruption of the *SODD* gene using homologous recombination-based gene targeting technology. However, to avoid the illegitimate recombination which is particularly frequent in *S. apiospermum*, we used as parent strain the *KU70Δ* mutant previously prepared from the *S. apiospermum* reference strain [25] by deletion of the *KU70* gene encoding the ATP-dependent DNA helicase II subunit 1, which leads to a defective non-homologous end-joining system [23]. Our experiments showed that the obtained double mutant exhibited increased susceptibility to oxidative stress, triazole antifungals, and macrophage-mediated killing, thus demonstrating that SodD may contribute to protect the conidia against oxidative stress.

## 2. Materials and Methods

### 2.1. Strain and Culture Conditions

This study was conducted on a *KU70Δ* mutant [23] derived from the wild-type strain *S. apiospermum* IHEM 14462, originally isolated from sputum sample from a CF patient and previously used for whole-genome sequencing [25]. Strains were maintained at 37 °C by weekly passages on potato-dextrose-agar plus chloramphenicol (PDA from Condalab, containing in g/L: dextrose, 20; infusion extract from potatoes, 4; chloramphenicol, 0.5; and bacteriological agar, 15), supplemented with 20 µg/mL phleomycin or 50 µg/mL hygromycin, for maintenance of the *KU70Δ* mutant or the double mutant *KU70Δ/SODDΔ*, respectively. All strains were stored at −80 °C as conidial suspensions in 20% glycerol. Conidia processed into any experiment were collected from 9-day-old cultures grown in PDA or yeast extract-peptone-dextrose (YPD containing in g/L: yeast extract, 10; peptone, 20; glucose, 20; and chloramphenicol, 0.5%) and resuspended in sterile water or saline before to be enumerating by hematocytometer counts.

### 2.2. Genomic DNA Extraction

Mycelium from 9-day-old cultures on PDA (supplemented or not with phleomycin or hygromycin) was ground in liquid nitrogen before resuspending the crushed material in lysis buffer (10 mM Tris-HCl; 1 mM EDTA; 2% Triton X-100; 1% SDS; 0.1 M NaCl, pH 8). Genomic DNA was extracted using the phenol-chloroform protocol (all reagents from Sigma-Aldrich, Saint-Louis, MI, USA) and precipitated with ethanol. DNA was then treated with RNase A 0.2 mg/mL and kept at 4 °C in Tris-EDTA buffer. Qubit 2.0 Fluorometer (Invitrogen, Carlsbad, CA, USA) was used to quantify DNA, and its integrity was verified by 1% agarose gel electrophoresis.

### 2.3. Disruption of the SODD Gene

**Figure 1:**
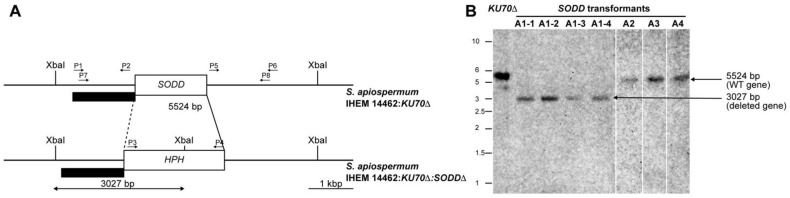
Production and Southern blot validation of the *KU70Δ/SODDΔ* mutant. (**A**): Restriction map of *S. apiospermum SODD* locus and strategy of construction of the disruption cassette. XbaI restriction sites are indicated, and the black box indicates the hybridization site of the probe (upstream region of the *SODD* gene). The size of the expected fragments is indicated by arrows. P1, P2, P3, P4, P5, P6, P7, and P8 correspond to the primers used for the construction of the disruption cassette. (**B**): Genomic DNA of the wild-type strain IHEM 14,462 (WT) and *SODD* transformants A1, A2, A3, or A4 were digested by XbaI. The 5.5-kbp band corresponding to the *SODD* wild-type locus was seen for the wild-type strain and *SODD* transformants A2, A3, and A4, but not for the A1 transformant, which showed a 3-kbp band demonstrating the disruption of the *SODD* gene by the *HPH* resistance gene.

For disruption of the gene encoding the GPI-anchored Cu,Zn-SOD, the cassette was prepared using the double-joint PCR procedure as described by Pateau et al. [29] with a few modifications. The selection marker used conferred hygromycin B resistance (*HPH*). The 5′ and 3′ flanking regions of the *SODD* gene were obtained from DNA from the wild-type strain by polymerase chain reaction (PCR) amplification using primers P1-*SODD* (forward) and P2-*SODD* (reverse), and primers P5-*SODD* (forward) and P6-*SODD* (reverse), respectively (Table 1, Figure 1A). As for the *HPH* gene, it was amplified from pAN7.1 plasmid using the forward and reverse primers P3-*SODD* and P4-*SODD* (Table 1), so that the amplified product overlapped the 5′ and 3′ flanking regions of the *SODD* gene. The three amplicons (i.e., 5′ flanking region, *HPH gene,* and 3′ flanking region) were then fused with a molar ratio of 1:3:1. A double-joint final PCR was carried out with primers P7-*SODD* (forward) and P8-*SODD* (reverse) (Table 1) to obtain the final disruption cassette. PCR amplifications were performed with Q5^®^ high-fidelity DNA polymerase (New England Biolabs, Beverly, MA, USA). Finally, the PCR construct was gel-purified using the Nucleospin^®^ gel and PCR clean-up kit (Macherey-Nagel, Düren, Germany).

The transformation was performed on protoplasts obtained from 24-h-old germ tubes as described by Turgeon et al. [30] and Liu and Friesen [31] with 5 µg of DNA. Germ tubes were collected by filtration on 20-µm-pore size Miracloth membranes and incubated at 37 °C for 3 h 30 under constant shaking (120 rpm) in OM/glucanex solution (1.2 M MgSO_4_, 10 mM Na_2_HPO_4_, 12.5 g/L glucanex, pH 5.8). Protoplasts were recovered by centrifugation in a Tris-HCl buffer (10 mM) containing 1.2 M sorbitol to maintain the osmotic pressure and then stored at 4 °C in the same buffer supplemented with 10 mM CaCl_2_. The cassette was integrated into protoplasts by heat shock in the presence of polyethylene glycol (PEG). Afterward, protoplasts were poured onto a soft agar medium (1 M sucrose, 0.2% yeast extract, 0.2% casaminoacids, 1.28% molten agar) in Petri dishes and incubated for 16 h at 37 °C before covering the plates with a layer of the abovementioned medium supplemented with 50 µg/mL hygromycin B. Cultures were incubated for 5 to 7 days at 37 °C and checked daily for transformants growing upward in the selection marker layer, which were then subcultured on PDA supplemented with 50 µg/mL hygromycin B.

### 2.4. Southern Blot Analysis

Transformants growing in the presence of hygromycin B were subcultured a couple of time to obtain monospore isolates, the genotype of which was then verified by Southern blot as previously detailed [29]. Genomic DNA was extracted and digested overnight with XbaI. After separation of the DNA fragments on agarose gel electrophoresis, gels were incubated successively in 0.25 N HCl, 1.5 M NaCl/0.5 M NaOH, and finally 0.5 M Tris-HCl pH 7.5/1.5 M NaCl. DNA fragments were then transferred on nylon membranes (Amersham Hybond^TM^-N^+^, GE Healthcare). After UV-crosslinking for 3 min, the gels were incubated overnight at 55 °C in the presence of the probe [a PCR product obtained from genomic DNA of the wild-type strain using P7-*SODD* as forward primer and P2-*SODD* as the reverse primer (Table 1, Figure 1A)] labeled with Illustra™ Shrimp alkaline phosphatase (GE Healthcare life sciences) according to the manufacturer recommendations for validation of the *KU70Δ/SODDΔ* double mutants. Finally, alkaline phosphatase was revealed by the addition of its substrate, and the membrane was imaged by chemiluminescence (LAS4000 GE Healthcare).

### 2.5. Susceptibility Studies

Multiple studies were conducted on the disruptant *KU70Δ/SODDΔ* and its parent strain *KU70Δ*.

*Sensitivity to stress conditions.* The viability of the conidia was analyzed in different stress conditions by spotting 5 μL of serial 10 fold dilutions of the conidial suspension (from 2 × 10^6^ to 2 × 10^1^ conidia/mL) on PDA plates incubated at different temperatures (28 °C, 37 °C, 42 °C), or on PDA plates supplemented with increasing concentrations of Calcofluor White (10ߝ160 µg/mL), NaCl (62.5 µMߝ1 mM), Congo Red (0.125ߝ0.5 µg/mL), sodium dodecylsulfate (SDS; 10ߝ320 µg/mL) or D-sorbitol (0.25ߝ2 M), which were incubated at 37 °C for 3 days. Presented data were obtained from three independent experiments, each comprising two technical replicates.

*Sensitivity to oxidative stress.* Unless otherwise specified, the sensitivity to oxidative stress was determined by monitoring the fungal growth in liquid medium by laser nephelometry as previously described [29,32]. Conidia were inoculated into YPD broth to give a final concentration of 10^5^ conidia/mL, and the obtained suspension was distributed into the wells of 96-flat bottom wells microtiter plates (300 μL per well). Assay wells were supplemented with one of the following chemicals: 25 µM menadione (Sigma-Aldrich), 0.5 mM diamide (Sigma-Aldrich), 1 mM cumene hydroperoxide (Sigma-Aldrich), or 8 μg/mL honokiol (Ak Scientific, Union City, CA, USA). Menadione was prepared as stock solution in dimethylsulfoxide (DMSO) so that solvent concentration in assay wells did not exceed 1% (*v*/*v*), whereas distilled water was used to prepare the stock solutions for the other chemicals. Microplates were incubated at 37 °C for 6 days in a laser-based microplate nephelometer (BMG Labtech, Ortenberg, Germany). During incubation, plates were subjected to shaking at 200 rpm for 5 min every 10 min, and growth was recorded after each shaking. Presented data were obtained from at least three independent experiments, each with triplicate wells. Growth curves expressing the relative nephelometric unit (RNU) as a function of time were generated using GraphPad Prism 6.0 (data not shown), and areas under curves (AUC) were calculated.

Because of its limited stability in liquid medium, we were unable to measure the effect of hydrogen peroxide using laser nephelometry. Sensitivity to H_2_O_2_ was therefore investigated by a disk diffusion method. Conidial suspensions were adjusted to 1 Mc Farland and spread onto YPDA plates using a spiral plater. Plates were left drying for 15 min in a biosafety cabinet. Sterile filter paper disks (10 mm in diameter) soaked with 49 or 98 mM H_2_O_2_ (100 µL per disk) were placed in the middle of the plates. Plates were incubated at room temperature for 24 h, then transferred to 37 °C. The diameter of the growth inhibition zones was assessed after a total of 72 h of incubation. At least 5 plates were made for each H_2_O_2_ concentration during each of three independent repetitions.

*Susceptibility to antifungal drugs.* Antifungal susceptibility testing was performed using the Etest method previously reported to deliver comparable results with the standard broth microdilution method regarding *Scedosporium* species [33]. Conidia prepared in saline were adjusted to 1 Mc Farland and streaked across the surface of an RPMI agar plate (bioMérieux, Marcy l’Etoile, France) using a spiral plater. Dishes were left drying for 15 min at 37 °C before applying the Etest strips (bioMérieux). The minimal inhibitory concentrations (MIC) were determined after 72 h of incubation at 37 °C; the MIC was defined as the lowest concentration at which the elliptical inhibition zone intercepts the strip. For triazole antifungals, microcolonies within the growth inhibition zone were overlooked. At least 5 Etest strips were used for each antifungal during each repetition.

### 2.6. Phagocytosis Assays

*Cell preparation and co-culture.* Monocytes were isolated from healthy human donors and differentiated to M1 phenotype-macrophages in complete RPMI 1640 (Lonza, Basel, Switzerland) containing 50 ng/mL of granulocyte-macrophage colony-stimulating factor (GM-CSF) as previously described [34]. Conidia were stained with 40 μg/mL of fluorescein isothiocyanate (FITC; Sigma-Aldrich) in phosphate-buffered saline 0.15 M pH 7.2 (PBS) for 30 min in darkness with constant shaking (120 rpm), washed twice with PBS, and enumerated. FITC-labeled conidia (5 × 10^5^ cells) were co-cultured with adherent M1 macrophages (cell ratio of 1:1) in a 6-well plate in a total volume of 500 μL of complete RPMI 1640. Macrophages were allowed to ingest conidia at 37 °C in 5% CO_2_ for 6 h. Later, plates were put on ice for 20 min to stop phagocytosis and processed into either the ingestion or the killing experiments. At least three independent replicates with technical triplicates in each were carried out for each strain.

*Ingestion assays.* Cells were lifted from wells by gentle mixing and transferred to Eppendorf tubes. The suspensions were washed twice with PBS and centrifuged at 500× *g* for 5 min to eliminate RPMI and non-adherent conidia. Macrophages were then marked with anti CD14 MAb (Miltenyi Biotec, Bergisch Gladbach, Germany) for 15 min at 4 °C, rinsed twice with PBS containing 1% bovine serum albumin and 0.1% sodium azide (PBS-BSA), and stained for viability with 7-aminoactinomycin D (7-AAD; Invitrogen). Cells were analyzed on FACSCanto II cytometer (Becton-Dickinson, Franklin Lakes, NJ, USA) to quantify the percentage of conidia ingested by macrophages. Data were gated to remove dead macrophages. A double-positive population (CD14+ FITC+) was considered to reflect macrophages with adherent and/or ingested conidia.

*Killing assays.* Non-adherent conidia were removed as described above. Afterward, adherent conidia were removed by discarding the supernatant after additional steps of washing with warm PBS and centrifugation at 500× *g* for 5 min. After checking the removal of adherent conidia under the microscope, the cell pellets were resuspended with 500 μL of water and incubated at 4 °C for 30 min to allow lysis of macrophages and release of intracellular conidia. After microscopic control of macrophage lysis, the obtained suspensions were centrifuged at 4600 rpm for 5 min to sediment released conidia. Macrophage debris was removed by treating the suspensions with DNase, RNase, and proteinase K (all from Invitrogen) as applied previously [35]. Each replicate included a negative control, reflecting the live conidia (macrophages-unexposed conidia), and a positive control referring to heat-killed conidia (85 °C, 30 min). If necessary, recovered conidia were kept at 4 °C until the next day. Conidia were then labeled with 25 μg/mL propidium iodide (PI; Sigma-Aldrich) for 30 min at room temperature in darkness before flow cytometry analysis. Both controls were used to set gates for the positivity of each marker. Conidia were considered live when they were only FITC-positive and dead in case of double-positivity (FITC+ PI+).

### 2.7. Transmission Electron Microscopy

Conidia collected from cultures grown on PDA were incubated at room temperature for 2 h with or without menadione and processed for transmission electron microscopy (TEM) as described by Ghamrawi et al. [28]. Sections were examined on JEM-1400 transmission electron microscope (Jeol, Paris, France). Only longitudinal sections of conidia (*n* = 20) were considered to evaluate the width, length, or thickness of the conidial wall.

### 2.8. Statistical Analysis

Statistical analysis was performed using GraphPad Prism 6.0. An unpaired t-test was used to check significant differences between the double mutant and its parent strain regarding the area under the growth curves in the presence of oxidative agents. Otherwise, the one-way analysis of variance Kruskal–Wallis test was run to evaluate differences between the two strains. Results were considered significantly different when *p* < 0.05.

## 3. Results

### 3.1. Generation of a SODD Deficient Mutant

Because of the high frequency of non-homologous recombination events in *S. apiospermum*, we used a mutant strain deficient for the non-homologous end joining (NHEJ) by disruption of the *KU70* gene, and the *SODD* gene was disrupted in this mutant by introducing the *HPH* resistance gene at the *SODD* locus. Culture on hygromycin-containing agar yielded only four colonies, corresponding to potential *SODD* disruptants (named A1, A2, A3, and A4) based on the hygromycin B resistance phenotype. Monospore isolates prepared from each colony were confirmed by PCR amplification of the *HPH* gene and finally analyzed by Southern blot after XbaI digestion of genomic DNA (Figure 1B). As expected for a correct gene disruption event, Southern blot analysis revealed a single 3-kb band for the transformant *KU70Δ/SODDΔ*-A1, whereas a 5.5-kb band was observed for the parent strain and the other transformants (Figure 1B).

### 3.2. Sensitivity to Temperature and Cell Wall Stressing Chemicals

Regarding the sensitivity to heat and chemical stress, no difference was observed between the parent strain (*KU70Δ* mutant) and the *KU70Δ/SODDΔ* double mutant in size or pigmentation of the colonies grown on PDA whatever was the temperature (28, 37, or 42 °C). Likewise, the disruption of the *SODD* gene did not affect the tolerance to osmotic agents (NaCl or sorbitol), SDS, or Calcofluor White. Conversely, the addition of Congo red to the culture medium resulted in a one-log growth inhibition compared to the parent strain (data not shown).

**Figure 2:**
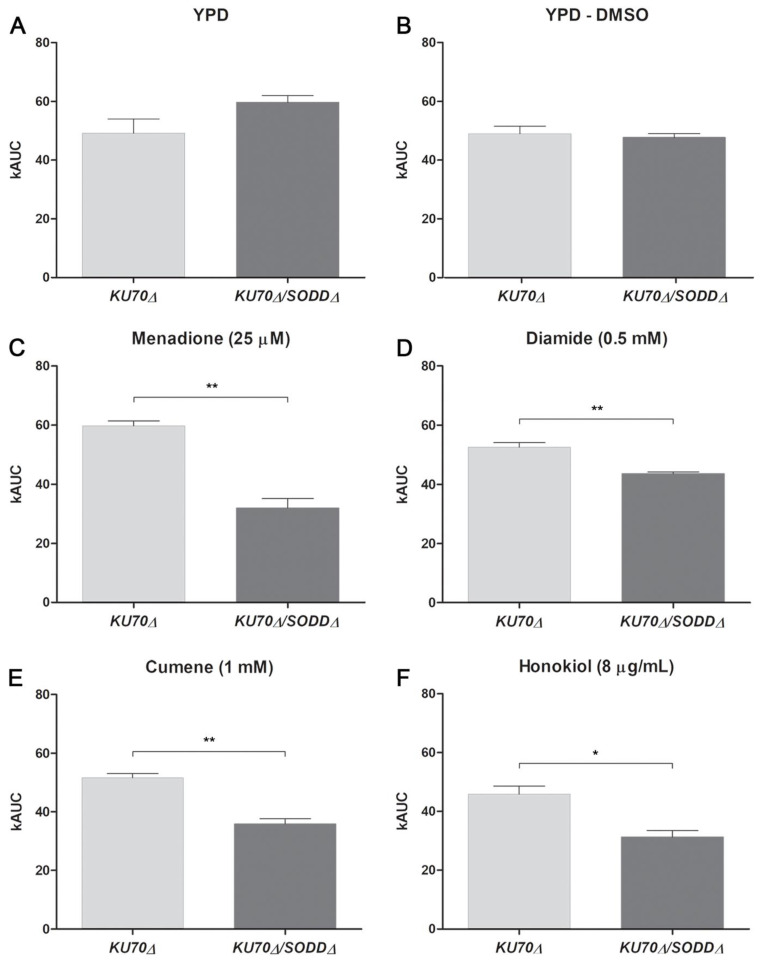
Laser nephelometry evaluation of the fungal growth. Growth of the *SODD* defective mutant (*KU70Δ/SODDΔ*) and its parent strain (*KU70Δ*) was monitored for 6 days by laser nephelometry in yeast extract-peptone-dextrose (YPD) broth in the presence of oxidizing agents. Positive controls were run in parallel: YPD alone (**A**) served as the control for all the tested agents except menadione, for which YPD plus dimethyl sulfoxide (DMSO) (**B**) served as the solvent control. (**C**): Menadione 25 μM. (**D**): Diamide 0.5 mM. (**E**): Cumene 1 mM. (**F**): Honokiol 8 μg/mL. The area under curve **(**AUC) values were calculated from curves expressing the relative nephelometric unit (RNU) as a function of time in h. Results correspond to the mean of triplicate determinations. Error bars correspond to the standard error of the mean. *, *p* < 0.05. **, *p* < 0.01.

The disruption of *SODD* gene did not affect the growth under normal conditions (YPD), as revealed by laser nephelometry which showed similar AUCs for the double mutant and its parent strain (Figure 2A). These results demonstrate that SodD is not essential for growth under normal conditions, osmotic stress, or varying temperatures, but it may be involved in the protection against some cell wall stress-inducing agents.

### 3.3. Growth under Chemically-Induced Oxidative Stress

As the main known function of SODs is to protect cells against ROS by breaking down O_2_^●−^ into H_2_O_2_, the growth of the double mutant was evaluated by laser nephelometry in YPD broth containing oxidant agents. Disrupting the *SODD* gene resulted in a significant increase in susceptibility to all the tested agents, which is reflected in decreased AUC values in comparison to its parent strain (Figure 2). In the presence of 25 µM menadione, the growth of the double mutant reached only 50% of that of its parent strain (Figure 2C), whereas it was not affected in the presence of the solvent alone (DMSO; Figure 2B). Likewise, the AUC obtained for the double mutant grown in the presence of 1 mM cumene or 8 μg/mL honokiol was about one-third lower (Figure 2E,F). The least significant difference in the AUC values between the strains was observed in the presence of 0.5 mM diamide (Figure 2D).

**Figure 3:**
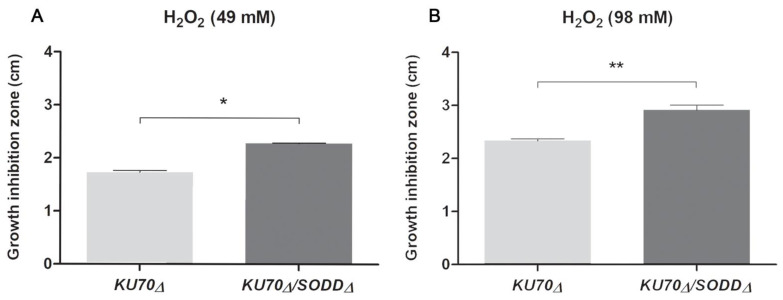
Growth-inhibition effect of H_2_O_2_-soaked disks on the *SODD* defective mutant (*KU70Δ/SODDΔ*) and its parent strain (*KU70Δ*). The diameters (cm) were measured after 72 h. (**A**): H_2_O_2_ 49 mM. (**B**): H_2_O_2_ 98 mM. Error bars correspond to the standard error of the mean. *, *p* < 0.05. **, *p* < 0.01.

Evaluation of the sensitivity to H_2_O_2_ by the disk diffusion method also suggested the involvement of SodD in the protection of the conidia against ROS. Diameter of the growth-inhibition zone was significantly higher for the double mutant compared to its parent strain (Figure 3A,B): using disks soaked with 49 mM or 98 mM H_2_O_2_, the diameters of the inhibition zones for the double mutant were 2.278 ± 0.019 cm and 2.913 ± 0.211 cm, respectively, whereas they were at least 20% lower for its parent strain (1.733 ± 0.057 cm and 2.340 ± 0.072 cm, respectively).

### 3.4. Susceptibility to Antifungals

The deficiency in the conidial-specific GPI-anchored SOD also affected the in vitro susceptibility to antifungals. Conversely to the susceptibility to fluconazole, amphotericin B, and the echinocandins, which were not impacted, the disruption of the *SODD* gene led to a significant decrease in the MIC values of isavuconazole, itraconazole, posaconazole, and voriconazole (Table 2).

### 3.5. Interactions with Phagocytes

Since SodD is specifically expressed at the conidial surface and potentially involved in their protection against ROS, we investigated whether the protein interferes with the phagocytosis process. Two parameters in the interaction between macrophages and the conidia, ingestion and killing, were evaluated using a flow cytometry-based technique that enabled robust measurement of each parameter. The ingestion percentage corresponds to the ratio between the number of live macrophages with adherent and/or ingested conidia to the total number of live macrophages multiplied by 100, while the killing was determined from the number of killed conidia among the total number of macrophage-released conidia.

**Figure 4:**
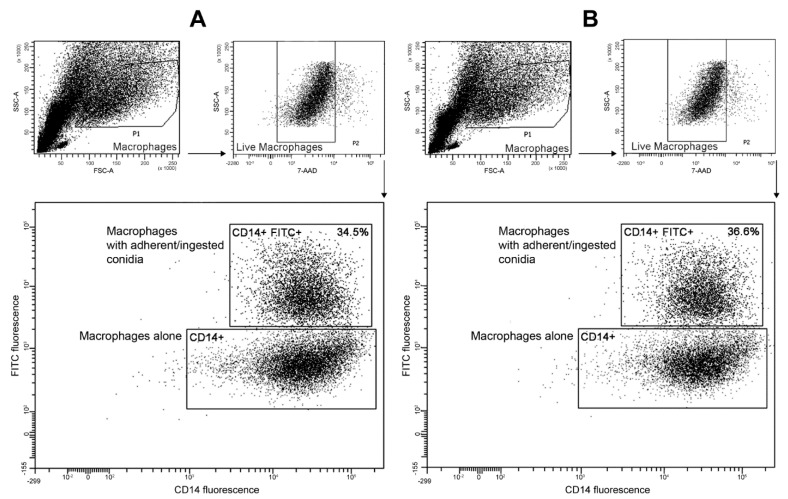
Flow cytometry analysis of macrophages ingesting conidia of *SODD* defective mutant (*KU70Δ/SODDΔ*) and its parent strain (*KU70Δ*). After 6 h of incubation with fluorescein isothiocyanate (FITC)-labeled conidia, CD14-labeled macrophages were selected apart from non-phagocytosed conidia (P1 population) on the histogram plotting side scatter (SSC) against forward scatter (FSC). Macrophages were then gated on the 7-aminoactinomycin D (7-AAD) channel to select live macrophages (P2 population) before plotting the cells against the fluorescence intensity in the scatter of CD14 vs. FITC fluorescence. Non-labeled conidia (FITC negative), as well as non-labeled macrophages (CD14 negative), were run to define the baseline of positive signal (not shown). Macrophages alone were only CD14+ signals, while macrophages with adherent or ingested conidia showed double-positive signals (CD14+ FITC+). (**A**): plots corresponding to the parent strain *KU70Δ*. (**B**): plots corresponding to the double mutant *KU70Δ/SODDΔ*. The percentage of ingestion displayed for each double-positive population refers to the ratio between the number of macrophages with adherent and/or ingested conidia and the total number of macrophages × 100. Presented data are representative of three independent experiments.

Concerning the ingestion analysis (Figure 4), because there were still non-adherent conidia in the suspensions and since macrophages are subjected to death after several hours of co-culture, we first selected the live macrophage population in the light scattered plot. Flow cytometry effectively differentiated between macrophages alone (CD14+) and macrophages with adherent/ingested conidia (CD14+ FITC+), which served in measuring the ingestion rate. The adherence and ingestion steps were not affected by disruption of the *SODD* gene since we found almost the same ingestion rate for the double mutant (Figure 4B) and its parent strain *KU70Δ* (Figure 4A).

**Figure 5:**
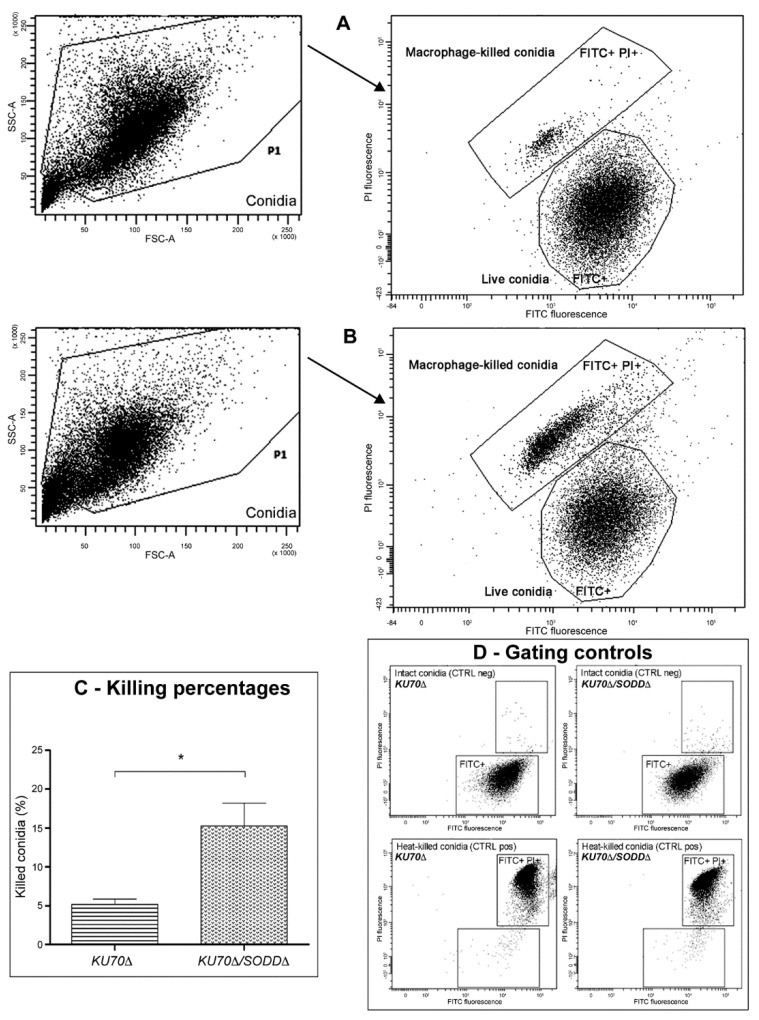
Flow cytometry analysis of the macrophage-mediated killing of conidia of *SODD* defective mutant (*KU70Δ/SODDΔ*) and its parent strain (*KU70Δ*). After 6 h of coincubation with macrophages, fluorescein isothiocyanate (FITC)-labeled conidia were released from macrophages by lysis of the cells, stained with propidium iodide (PI), and analyzed by flow cytometry. On the forward scatter (FSC) vs. side scatter (SSC) histogram plot, conidia with values lower than 50 were excluded from selection. Conidia were then gated on the scatter plot of FITC against PI fluorescence to define populations: FITC+ signals correspond to live conidia, while double-positive signals (FITC+ PI+) relate to macrophage-killed conidia. The baseline of positivity of each marker was defined using non-labeled conidia (not shown). (**A**): plots corresponding to the parent strain *KU70Δ.* (**B**): plots corresponding to the double mutant *KU70Δ/SODDΔ*. The plots are derived from one experiment representative of the other experiments. (**C**): the percentage of killing was defined as the number of macrophage-killed conidia to the total number of macrophage-released conidia × 100; killing percentage data were collected from three independent experiments; error bars correspond to the standard error of the mean; *, *p* < 0.05. (**D**): negative control (fresh live conidia) and positive control (heat-killed conidia) were run to define population borderlines (please see Results section for further explanation).

For the killing assay (Figure 5), all events should theoretically correspond to intracellular conidia that have been released from macrophages. However, despite treatment of the cell lysate with DNase, RNase, and proteinase K, it remained necessary to eliminate all small events that probably refer to cell debris. Thus, events with low forward scatter (FSC) and side scatter (SSC) values were excluded. Fresh live conidia (FITC+ PI-) and heat-killed conidia (FITC+ PI+) were run as controls to define the area of the correspondent populations on the FITC vs. PI histogram plot (Figure 5D). However, although still within the positivity zone, the conidia that have been killed intracellularly by macrophages exhibited lower signals for FITC and PI fluorescence (Figure 5A,B). It is likely that heat-killing and macrophage-mediated killing of the conidia do not have similar impacts on the conidial surface fluorescence and membrane permeabilization; heat-killing results in a rapid membrane permeabilization allowing an important PI uptake, while during the macrophage-mediated killing, the slow membrane damaging leads to a gradual loss of FITC-labelled surface proteins and a lesser PI uptake. Nevertheless, the double-positive population (FITC+ PI+) of macrophage-killed conidia was denser and broader for the double mutant *KU70Δ/SODDΔ* (Figure 5B) than for its parent strain *KU70Δ* (Figure 5A); a significant difference was seen between the two strains in the killing rate with values of 15.25 ± 5.94% for the double mutant vs. 5.2 ± 1.314% for its parent strain (Figure 5C). These results, which were reproducible even when different donor-derived macrophages were used, support the involvement of the GPI-anchored cell wall SodD in protecting the conidia against macrophage-mediated killing.

### 3.6. Morphological Features of the Conidia and Ultrastructure of Their Cell Wall

**Figure 6:**
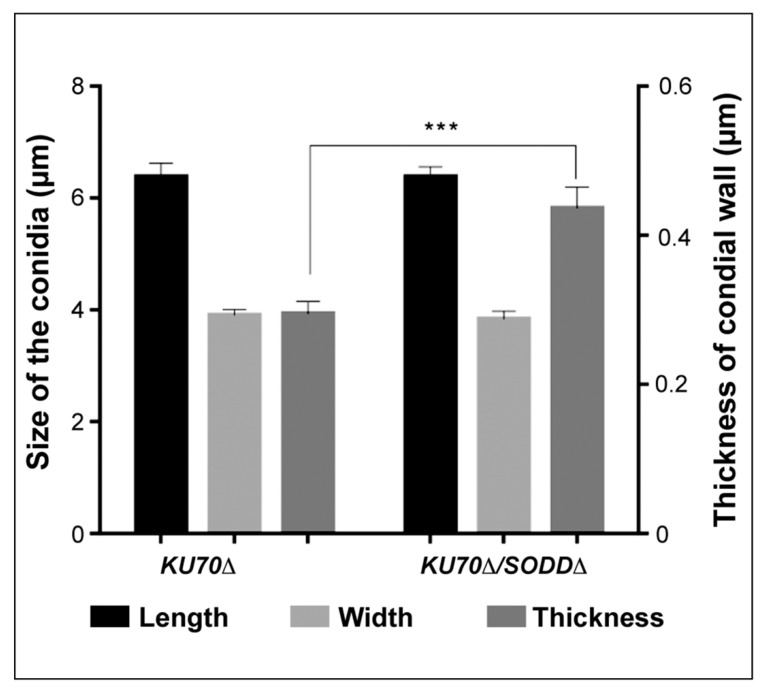
Length, width and thickness of the cell wall of the conidia of the SodD defective mutant (*KU70Δ/SODDΔ*) and its parent strain (*KU70Δ*) as determined by TEM examination. Significant differences were seen in the thickness of the conidial wall between the SodD defective mutant *KU70Δ/SODDΔ* and its parent strain *KU70Δ* (*** *p* = 0.0002), whereas length and width of the conidia were unchanged.

The higher susceptibility of the double mutant to Congo red known to bind to chitin prompted us to investigate the ultrastructure of the conidial wall (Figure 6). No differences were found in the width or length of the conidia. Conversely, whereas the conidial wall of the wild-type strain and the *KU70Δ* mutant exhibited a similar ultrastructure with a very thin electron-dense outer cell wall layer and a thicker inner layer, a thickening of the inner cell wall layer was observed in conidia of the *KU70Δ/SODDΔ-A1* double mutant.

## 4. Discussion

Although ranking second among the filamentous fungi colonizing the CF airways, the pathogenic mechanisms that allow *Scedosporium* species to establish within the respiratory tract and to cause infections, remain poorly understood. Colonization of the CF bronchial mucus is thought to result from inhalation of some airborne conidia and their entrapment into the viscous mucus. Therefore, the mechanisms allowing the conidia to evade the host immune response, mainly represented by the oxidative burst response of phagocytes and the production of ROS, are essential for a successful colonization process. Recently, we showed that *Scedosporium* species were able to germinate in oxidative stress conditions [36]. In addition, analysis of the *S. apiospermum* genome revealed the presence of 33 genes encoding putative enzymes possibly involved in the degradation of ROS or RNS [26]. Some of these genes were found to be overexpressed in response to the exposure of hyphae to oxidative stress or after co-culture with lipopolysaccharide-activated macrophages or neutrophils [27]. Nevertheless, the design of these experiments did not allow to investigate the role of genes encoding conidial-specific enzymes, such as SodD, which is a GPI-anchored cell wall protein specifically found on the conidia [26,28]. The present study, therefore, aimed to determine the role of this enzyme. While it is not essential for the growth of *S. apiospermum* IHEM 14,462 *KU70Δ*, SodD was shown to protect the conidia from damages caused by oxidizing agents.

Previously Pateau et al. [29] produced from a wild-type strain of *S. aurantiacum* defective mutants for the cytosolic Cu,Zn-SOD SodC and showed a surprising frequency of homologous recombination as high as 30%. Other results from our laboratory suggested a markedly lower frequency in *S. apiospermum*, consistently to those reported in the literature for other filamentous fungi (i.e., <3%) [37]. A mutant strain deficient for the non-homologous end joining system by deletion of the *KU70* gene, therefore, was used as parent strain as previously done for the *SidD* gene [23]. This strategy which is the gold standard to increase the frequency of homologous recombination in filamentous fungi [38], allowed us to disrupt the *SODD* gene in *S. apiospermum*. In our study, we have been limited by the very few resistance markers that can be applied to *Scedosporium* species since they are naturally resistant to many drugs. Currently, only two are effective, phleomycin that was used for engineering of the *KU70Δ* strain and hygromycin B, the one used for the selection of the defective strain *KU70Δ/SODDΔ.* No other selection markers were available, and further genetic tools should be developed to allow the production of complemented strains in *Scedosporium* species.

The role of SODs in the evasion of fungi to the oxidative stress response of phagocytic cells is well established for numerous human pathogens [29,39,40,41,42,43,44,45,46,47,48,49,50,51]. The degradation of ROS by microorganisms is an essential process for successful infection, and SODs are the first-line defense against oxidative stress. SODs are mainly intracellular (cytosolic or mitochondrial), and few extracellular SODs have been described to date. SOD defective mutants were obtained in *A. fumigatus* [46], *C. albicans* [41,47], *C. neoformans* [42], *Histoplasma capsulatum* [48,50], *Paracoccidioides brasiliensis* [51], and recently in *S. aurantiacum* [29]. Here, we have successfully constructed a defective mutant for the *SODD* gene encoding a GPI-anchored SOD in *S. apiospermum*. This enzyme does not only belong to a particular class of extracellular SODs containing a signal peptide at the N-terminus and a GPI-anchor binding site [26], but it is also specifically expressed at the conidial surface [28]. GPI-anchored proteins are known to participate in other fungi to morphogenesis, stress resistance, and virulence [52,53,54,55].

While the role of cytosolic Cu,Zn-SODs in pathogenesis has been extensively investigated in *C. albicans* [41,45]*, C. neoformans* [42,56,57], and *A. fumigatus* [46,58,59], little information is available regarding GPI-anchored SODs. Recently, it was shown that the GPI-anchored Sod2 from the phytopathogenic fungus *Puccinia striiformis* suppressed ROS accumulation during infection and was essential for its virulence [60]. Regarding human pathogenic fungi, three GPI-anchored SODs, i.e., Sod4p, Sod5p, and Sod6p, were described in *C. albicans* [45], and one of them, Sod5p, was shown to be involved in the protection of blastoconidia against oxidative stress and in virulence [43,45,47,49,61]. Likewise, *P. brasiliensis* and *H. capsulatum SOD3* genes, two orthologs of *S. apiospermum SODD* gene [26]*,* were shown to be involved in virulence [48,51]. For example, the disruption of the *SOD3* gene in *P. brasiliensis* resulted in attenuated virulence in mice conversely to the disruption of the *SOD1* gene encoding a cytosolic Cu,Zn-SOD [51]. Our results suggest that SodD plays an important role in protecting *S. apiospermum* conidia against superoxide radicals. As with *P. brasiliensis* Sod3, SodD proved not essential for fungal growth; still, its defect results in increased sensitivity to chemically-induced oxidative stress as attested by the reduced growth of the double mutant exposed to various ROS-induced agents or to H_2_O_2_ compared to its parent strain.

Since SodD is localized to the cell envelope, a question arises on how the enzyme protects the fungus from endogenous O_2_^●−^-inducing agents such as menadione and honokiol. Menadione is essentially an intracellular O_2_^●−^-inducer; however, the addition of Sod1 to the incubation buffer was shown to protect *Saccharomyces cerevisiae* cells from the cytotoxic effect of menadione [62], which is in line with our findings of SodD playing a protective role against exogenous oxidative stress imposed by menadione. Honokiol is a natural biphenolic compound that disrupts mitochondrial respiration leading to intracellular ROS accumulation, mainly O_2_^●−^. Interestingly, all *SOD* genes harbored by *C. albicans* were upregulated in response to honokiol, including three GPI-anchored ones [63]. One may hypothesize a defect in membrane trafficking of SodD following such stress. Primary findings on *Fusarium oxysporum* Sod5 (a GPI-anchored Cu,Zn-SOD) support this hypothesis, showing that the Sod5 subcellular localization is altered under unfavorable environmental conditions [64]. Finally, the peroxidase activities exhibited by all SOD types can explain the increased sensitivity of our mutant defective in *SODD* gene to H_2_O_2_ [65]. While the glutathione system is required for tolerance to cumene hydroperoxide and diamide, it is still unknown how SodD contributes to such resistance. Further studies are needed to unravel the mechanisms by which this enzyme fights various oxidizing agents.

In *C. albicans*, *A. fumigatus,* and *Cr. neoformans*, SODs have been suggested to contribute to tolerance to antifungal drugs, including imidazoles and triazoles [66,67,68,69,70]. Here we showed the contribution of *S. apiospermum* SodD in tolerance of the fungus to isavuconazole, itraconazole, posaconazole, and voriconazole, and to a lesser extent to amphotericin B and micafungin. There is limited information linking the antifungal activity of azole drugs and oxidative stress. Nevertheless, there is now accumulating evidence for an increased production of endogenous ROS induced by the antifungal drugs [66,67,68,69,70]. Likewise, treating *C. albicans* or *Candida dubliniensis* blastoconidia with fluconazole elicited increased SOD and catalase production [71]. As suggested above, one may speculate a defect in membrane trafficking of SodD following exposure to antifungal drugs and the contribution of this enzyme to the degradation of ROS accumulating in mitochondria.

In order to prove the involvement of *S. apiospermum* SodD in the interaction of the conidia with macrophages, a flow cytometry based-protocol was developed that enabled the differentiation between the different cell populations. To the best of our knowledge, this is the first time such a protocol has ever been used on *Scedosporium* species or *Lomentospora prolificans* to investigate the phagocytosis process. The involvement of GPI-anchored SODs in recognition of fungal pathogens by phagocytic cells has never been documented, and as expected, the adherence and ingestion steps of *S. apiospermum* conidia were not affected by disruption of the *SODD* gene. Indeed, the ingestion rates were obviously similar for the mutant defective in the *SODD* gene and its parent strain.

By contrast, some fungal GPI-anchored SODs were shown to contribute to surviving the ROS-dependent host killing. For instance, it was shown that *C. albicans* Sod4 and Sod5 were essential for protecting the blastoconidia against ROS-dependent macrophage killing [45]. Likewise, in *H. capsulatum*, the disruption of *SOD3* gene also led to a decreased viability of yeast cells exposed to neutrophils and macrophages, and in vivo experiments demonstrated that Sod3 promotes *Histoplasma* virulence in a murine model of disseminated infection [48]. Consistent with these findings, we showed *S. apiospermum* GPI-anchored SodD to be relevant in protecting the conidia against the intracellular macrophages-mediated killing. Disruption of *SODD* gene led to an increased killing of ingested conidia by macrophages, although their germination was not affected. Indeed, no differences were seen in the latency period between the double mutant and its parent strain in laser nephelometry study of the growth under control conditions, e.g., without any oxidative chemical (Figure S1).

An indirect role of the enzyme in susceptibility of the conidia to killing by macrophages should also be considered because of possible biochemical changes of the cell wall that would have been caused by disruption of the *SODD* gene. TEM analysis of the conidia revealed for the *KU70Δ/SODDΔ* double mutant a thickening of the cell wall, associated with an increased susceptibility to Congo red, whereas tolerance to Calcofluor White was not affected. Discrepancies in the susceptibility to these cell wall disturbing agents have already been reported. For example, the *SKN7* gene contributes to cell wall integrity in *A. fumigatus*, and its disruption does not modify the susceptibility to Congo red, whereas the *SKN7Δ* mutant is more resistant to Calcofluor White compared to its parent strain [72]. Calcofluor White is known to bind to chitin, whereas Congo red may also interfere with glucans as described in *S. cerevisiae* [73]. Contrasting with other fungi, glucans have been detected in the cell wall of *Scedosporium* species, but mainly alpha-glucans and only a low level of beta-glucans [74]. Nevertheless, the increased susceptibility to Congo red resulting from disruption of the *SODD* gene suggests a defect in cell wall integrity. Studies in *S. cerevisiae* and *A. fumigatus* have demonstrated the importance of some GPI-anchored proteins for cell wall integrity [52,75] and it has been demonstrated that, when the cell wall integrity is compromised, fungal cells respond by increasing cell wall biosynthesis [76]. Therefore, further studies are needed to define the impact of *SODD* gene disruption on the composition of the cell wall.

## 5. Conclusions

In conclusion, an *S. apiospermum* NHEJ deficient strain was used here to generate a defective mutant for the *SODD* gene. This allowed us to point out the role of this GPI-anchored SOD in protecting the conidia against various chemically-induced oxidative stresses. The loss of SodD also increased the susceptibility to triazoles, highlighting another function of this enzyme in contributing to tolerance to azole drugs. In addition, a flow cytometry-based protocol was developed that enabled the assessment of conidia-macrophage interactions. SodD does not affect the recognition of conidia, nor their adherence to and ingestion by macrophages, but plays a relevant role in escaping macrophage-mediated killing. Nevertheless, one cannot disregard an indirect role of this enzyme since TEM analysis of the conidia of the double mutant revealed ultrastructural changes in the cell wall with a marked thickening of the inner cell wall layer. Complementary studies will be conducted on the *SODD* defective mutant and its parent strain to definitively demonstrate the surface location of SodD, and to the analyze the impact of gene disruption on the biochemical composition of the cell wall and on virulence in animal models of *Scedosporium* infections.

## Figures and Tables

**Table 1:** List of primers used for construction of the cassette for *SODD* gene disruption.

Primer Name and Use	Sequence 5′→3′	Tm (°C)	Size of the Amplicon (bp)
*PCR amplification of the 5′ flanking region of* SODD			
P1-*SODD*	ATTCATAGACTCAATAATTAGAACTCGACT	64	925
P2-*SODD*	**TCGTGAATCTTTTACCAGATCGGAAGCAAT**AAATGTAATTTATCTCTTTCAATCCCAAGC	64	
*PCR amplification of the hygromycin B resistance gene*			
P3-*SODD*	GCTTGGGATTGAAAGAGATAAATTACATTT**ATTGCTTCCGATCTGGTAAAAGATTCACGA**	68	2633
P4-*SODD*	AATTGATTCTTGTCGATCATTAATTTGGTC**ATCAGAGCAGATTGTACTGAGAGTGCACCA**	68	
*PCR amplification of the 5′ flanking region of* SODD			
P5-*SODD*	**TGGTGCACTCTCAGTACAATCTGCTCTGAT**GACCAAATTAATGATCGACAAGAATCAATT	64	901
P6-*SODD*	GACGTTGTATATATATCCTGGAAGAATCTT	64	
*Fusion of the amplicons*			
P7-*SODD*	GAAACGCCCGACTAGTTAAATC	64	4448
P8-*SODD*	CTGCAAATGCCAAATTCCAA	64	

Sequences that are indicated in bold font correspond to homologous sequences overlapping with the resistance marker.

**Table 2:** In vitro susceptibility to antifungals.

Antifungal	Parent Strain (*KU70Δ* Mutant)		*KU70Δ/SODDΔ* Double Mutant		*p*-Value
	MIC Mean (µg/mL)	SD	MIC Mean (µg/mL)	SD	
Isavuconazole	>32	ND	0.142	0.093	<0.05
Itraconazole	0.552	0.187	0.064	0.000	<0.05
Posaconazole	0.476	0.053	0.131	0.035	<0.05
Voriconazole	0.251	0.146	0.018	0.004	<0.05
Fluconazole	>256	ND	>256	ND	NS
Amphotericin B	5	2	3.9	1.597	NS
Caspofungin	>32	ND	>32	ND	NS
Micafungin	0.082	0.016	0.113	0.048	NS

Antifungal susceptibility testing was performed on the double mutant and its parent strain using the Etest strip method on RPMI-dextrose agar plates. Differences in minimum inhibitory concentration (MIC) values between strains were considered significant when *p* < 0.05. SD, standard deviation; ND, not determined; NS, non-significant.

## Data Availability

All data generated or analyzed during this study are included in this published article. The raw data supporting the conclusions of this article will be made available by the authors to any qualified researcher.

## Supplementary Materials

The following are available online at https://www.mdpi.com/article/10.3390/jof7070575/s1, Figure S1: Growth kinetics of the double mutant *KU70**Δ/SODD**Δ* and its parent strain *KU70**Δ* under control conditions. Growth was monitored by laser nephelometry in yeast extract-peptone-dextrose broth without any oxidative chemical.
