# Supplementary material for: The Glycosylphosphatidylinositol-Anchored Superoxide Dismutase of Scedosporium apiospermum Protects the Conidia from Oxidative Stress
Source: J Fungi (Basel). 2021 Jul 19;7(7):575. doi: 10.3390/jof7070575 (PMC8304446; doi:10.3390/jof7070575)
Supplement: Supplementary file 1 [file jof-07-00575-s001.zip › jof-1278335-supplementary.pdf]

The glycosylphosphatidylinositol-anchored superoxide dismutase of *Scedosporium apiospermum* protects the conidia from oxidative stress

Cindy Staerck, Hajar Yaakoub, Patrick Vandeputte, Julie Tabiasco, Charlotte Godon, Amandine Gastebois, Sandrine Giraud, Thomas Guillemette, Alphonse Calenda, Yves Delneste, Maxime Fleury and Jean-Philippe Bouchara

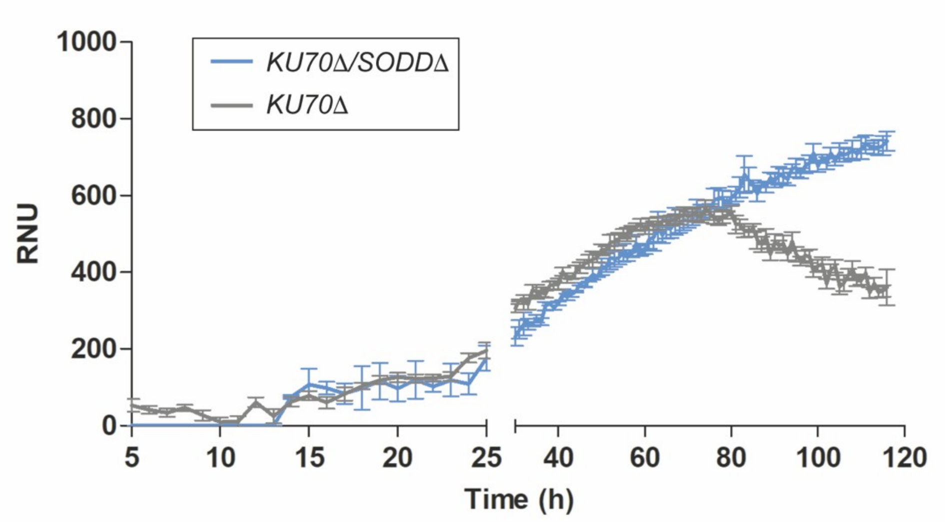

Figure S1: Growth kinetics of the double mutant *KU70Δ/SODDΔ* and its parent strain KU70*Δ* under control conditions. Growth was monitored by laser nephelometry in yeast extract-peptone-dextrose broth without any oxidative chemical.
